# Depression and Coronary Artery Disease—Where We Stand?

**DOI:** 10.3390/jcm14124281

**Published:** 2025-06-16

**Authors:** Anastasios Apostolos, Konstantinos Konstantinou, Nikolaos Ktenopoulos, Panayotis K. Vlachakis, Ioannis Skalidis, Grigorios Chrysostomidis, Vasileios Panoulas, Konstantinos Tsioufis

**Affiliations:** 1 First Department of Cardiology, Medical School, National and Kapodistrian University of Athens, Hippocration General Hospital of Athens, 11527 Athens, Greece; anastasisapostolos@gmail.com (A.A.); nikosktenop@gmail.com (N.K.); vlachakispanag@gmail.com (P.K.V.); ktsioufis@gmail.com (K.T.); 2 Department of Cardiology, Guy’s and St Thomas’ NHS Foundation Trust, Harefield Hospital, London UB9 6JH, UK; v.panoulas@rbht.nhs.uk; 3 Institut Cardiovasculaire Paris Sud (ICPS), 91300 Massy, France; skalidis7@gmail.com; 4 Second Department of Adult Cardiac Surgery, Onassis Cardiac Surgery Center, 17674 Athens, Greece; gregorychrisostomidis@gmail.com

**Keywords:** depression, stress, coronary heart disease, review

## Abstract

Coronary artery disease (CAD) and mental health disorders, particularly depression and anxiety, exhibit a complex, bidirectional relationship that adversely influences clinical outcomes and mortality. Mental illnesses account for approximately 8 million deaths annually, while cardiovascular diseases, including CAD, contribute to about 17 million deaths, with CAD alone responsible for one-third of deaths among individuals aged ≥35 years. This review offers a structured synthesis of current knowledge focusing on the (1) epidemiology, emphasizing the reciprocal risk between CAD and psychiatric conditions; (2) pathophysiological insights, including inflammation, neurohormonal dysregulation, platelet hyperactivation, and shared genetic determinants; and (3) therapeutic approaches, encompassing pharmacological management, psychotherapeutic interventions, and integrated care models. Selective serotonin reuptake inhibitors (SSRIs) remain the pharmacologic agents of choice in patients with CAD and depression due to their favorable cardiac profile, while cognitive behavioral therapy (CBT) offers psychological benefit. However, evidence for mortality reduction remains limited. Emerging research highlights the importance of biomarker-driven care, gut–brain–heart axis modulation, and AI-enabled clinical integration.

## 1. Introduction

Mental health disorders and cardiovascular diseases (CVDs) are leading causes of morbidity and mortality worldwide, creating significant challenges for public health systems. About 8 million deaths per year are associated with psychiatric illnesses, with individuals suffering from these conditions demonstrating a notably increased risk of mortality compared with the general population [1]. This increased risk is associated with a combination of behavioral, biological, and social factors, which also complicate the management of other long-lasting diseases. Moreover, CVDs remain the leading cause of death globally, as they account for approximately 17 million deaths per year. Despite advancements in medical therapies and revascularization techniques that have contributed to declining mortality rates in some regions, CVD remains a contemporary issue and coronary artery CAD remains the cornerstone, because it is responsible for nearly one-third of all deaths among individuals aged 35 years and older [2].

The relationship between mental health disorders and CVDs is bidirectional and complex. Cardiovascular patients are at increased risk of developing mental health disorders due to the psychological and physical toll of living with chronic illness [3,4]. Vice versa, individuals with psychiatric conditions, particularly depression and anxiety, exhibit an increased risk of developing coronary artery disease (CAD). This bidirectional link highlights the need for a comprehensive understanding of the interplay between mental health and cardiovascular health, as these two domains often exacerbate one another, leading to poor outcomes and significant healthcare burdens [5].

Although the interconnection between mental health disorders and CAD is well-established, the exact mechanisms explaining this relationship are not totally understood. Behavioral factors, like smoking, sedentary lifestyles, and poor adherence to medical therapies, as well as biological pathways, including chronic inflammation and autonomic dysregulation, play pivotal roles. The shared risk factors and overlapping pathophysiological processes between these conditions necessitate an integrated approach to their prevention and treatment.

Our review aims to present a structured synthesis of current knowledge focusing on the (1) epidemiology, emphasizing the reciprocal risk between CADs and psychiatric conditions; (2) pathophysiological mechanisms, including inflammation, neurohormonal dysregulation, platelet hyperactivation, and shared genetic determinants; and (3) therapeutic approaches, encompassing pharmacological management, psychotherapeutic interventions, and integrated care models.

## 2. Epidemiological Associations

Coronary artery disease, either presented as acute (ACS) or chronic (CCS), remains a stressful event, mainly when affecting younger patients. A significant proportion, varying between 19 and 66%, of the total number of patients with a previous MI present with mental disorders, mainly depression or anxiety [6]. Most of the symptoms are presented for the first time during the hospitalization of the patient, due to the ACS [7]. This percentage could be further increased when the patients undergo a percutaneous or even surgical intervention [8,9].

Several studies have supported that women with CAD are more prone to developing depression. Among women, depression is about twice as prevalent as in men; the same goes for female and male patients with CAD [10,11]. Depression is more frequent in young women with a previous MI; interestingly, about 50% of the women under 60 years with a previous MI suffer from major depression [12].

Depression affects about 10% of population [13], being the most frequent mental health disorder diagnosed in the general population [14,15]. However, this percentage might be underestimated; a meta-analysis showed that only half of patients with major depression are identified when the appropriate screening tests are applied [16]. Taking this into consideration, it is understandable that the proportion of patients with mild or moderate depression being undiagnosed would be even greater.

It is well-established that patients with depression are under higher risk of developing CAD and vice versa. Several studies [17,18] and multiple meta-analyses have identified this relationship [19,20,21]. In the Epidemiologic Catchments Area (ECA) study, patients with a history of dysphoria or depression had about a 4.5 times higher relative risk of suffering from an acute MI compared with non-depressed patients, adjusted for the conventional cardiovascular risk factors [22].

This phenomenon is better documented and supported in female than male patients. Among women, depression increases the risk of CAD significantly from 30% to 200% and this risk varies among different depression stages [23]. It is noteworthy that major depression or a previous suicide attempt was related with a 15-fold increased risk of CAD among women but only a 3.5-fold increased risk in men [24]. Against the same background, another study from the USA showed that women younger than 40 years diagnosed with depression had a six-fold higher risk of CAD compared with women without depression; meanwhile no significant difference was observed in men [25].

Interestingly, even patients meeting some but not all of the criteria required for depression had a higher risk of developing ACS or CCS, compared with those without any criteria [13,26]. Such findings show that there is a linear association between the severity of depression and the risk of developing CAD, and that such patients should not be overlooked in the terms of cardiovascular risk. Depression presents in heterogeneous clinical forms, each potentially exerting a distinct impact on cardiovascular risk. Consequently, each subtype has different impact on CAD development. Treatment-resistant depression, somatic symptoms, and onset after ACS are independently associated with ACS and CCS [27].

Depression is not only associated with a higher risk of acute and chronic coronary syndrome, but it has been associated with more frequent complications of CADs. More specifically, patients with depression presented more frequently with complications, including recurrent ischemia or infarction, heart failure, and arrhythmias, compared with those who were non-diagnosed [28].

It is noteworthy that the American Heart Association has identified depression as an independent risk factor for developing acute coronary syndrome (ACS) [29]. The Translational Research Investigating Underlying Disparities in Acute Myocardial Infarction Patients’ Health Status (TRIUMPH) trial evaluated the relationship between CAD and depression by comparing patients with acute MI and depression who received treatment for their depression versus those who did not. It included a total of 4062 patients and showed that those with untreated depression had about 70–90% higher one-year mortality, compared with patients without depression or with treated depression [30]. Therefore, the authors recommended that depression screening should be performed in every patient presented with ACS, so as to further reduce the burden of CAD. Nevertheless, this screening should not only be applied in patients with ACS, but also in patients with CCS. A recent study found that patients with a diagnosis of depression at any point following a diagnosis of CAD had a two-fold higher risk of all-cause death than those patients with CAD and no diagnosis of depression. Impressively, depression was a stronger predictor of mortality than any other risk factor or comorbidity [31]. Thus, screening for depression and the evaluation of its severity, when is already diagnosed, should be of prime interest.

The Enhancing Recovery in Coronary Heart Disease (ENRICHD) trial was a randomized, controlled trial, which included a total of 2481 patients with a recent MI and diagnosed with untreated minor or major depression. Patients were randomized to receive usual medical care or cognitive behavior therapy (CBT) with serotonin reuptake inhibitor (SSRI) treatment, when it was clinically necessary. The study failed to show difference in hard endpoints, like mortality or recurrent MI, but demonstrated benefit in the interventional arm, in the terms of depression symptoms and social isolation [32].

Considering that depression has been associated with poorer results of patients with recent MI, further studies exploring the role of antidepressive drugs have been conducted. The Canadian Cardiac Randomized Evaluation of Antidepressant and Psychotherapy Efficacy (CREATE) trial explored the role of citalopram and interpersonal psychotherapy in 284 patients with CAD and major depression. Citalopram showed clear benefit compared with a placebo; on the other hand, interpersonal psychotherapy did not offer any additional benefits compared with standard clinical management per se [33]. Therefore, pharmacotherapy, with either citalopram or sertraline, should be the first step in the management of patients with concomitant CAD and depression, instead of psychotherapy.

## 3. Behavioral and Clinical Factors Contributing to Poor Outcomes

Beyond biological mechanisms, several behavioral and health-system factors contribute significantly to the worsened prognosis observed in patients with coexisting depression and CAD. Depression is often accompanied by apathy, anhedonia, and a diminished sense of urgency, all of which might delay the recognition and reporting of cardiac symptoms. Several studies have shown that depressed patients are more likely to present late after the onset of ACS symptoms, reducing the window for optimal reperfusion therapies [29,32]. Patients with depression demonstrate markedly lower adherence to secondary prevention strategies, including antiplatelet therapy, statins, beta-blockers, ACE inhibitors, and possibly, heart failure treatment [34,35]. Depression has been identified as an independent predictor of medication non-compliance, leading to increased rates of rehospitalization and adverse cardiovascular events [36].

Furthermore, women with depression may face additional barriers due to the atypical presentation of CAD symptoms, such as fatigue, dyspnea, or gastrointestinal discomfort, which are more likely to be misattributed to non-cardiac causes. These diagnostic delays, compounded by under-recognition of depression in female patients, contribute to worse outcomes [23,37].

While SSRIs are generally considered safe for cardiac patients, some agents have been associated with weight gain and, in rare cases, altered glycemic control, potentially accelerating insulin resistance and metabolic syndrome. Current literature data support that these effects may be agent-specific rather than class-wide. For example, paroxetine has been associated with modest weight gain over long-term use, whereas sertraline and escitalopram are metabolically more neutral [38,39].

## 4. Pathophysiological Mechanisms

Several pathophysiological mechanisms have been proposed and explored for determining the association between CAD and depression. (Table 1) The hyperactivation of the sympathetic system is one of the most described possible mechanisms that might explain the relationship between depression and CAD. In patients suffering from depression, sympathetic outflow is significantly increased, due to the negative stress effects of catecholamines on the cardiovascular system and platelets. The association with catecholamine is well-supported; increased urinary catecholamines levels are linked with depressive emotions and high levels of norepinephrine and low of platelets serotonin are associated with MI and depression [6,40].

Additionally, it has been found that stress exposure can dysregulate excretion and the synthesis and action of noradrenalin, serotonin, dopamine, and cortisol [41,42,43,44]. Therefore, these alterations could lead to production of specific inflammatory cytokines, such as IL-1, IL-6, and TNF-a, and enhancement of inflammation, all known risk factors for the development of atherosclerosis and CAD [45,46,47]. C-reactive protein (CRP), a typical marker of systemic inflammation, is increased in patients diagnosed with depression [48]. In addition, numerous studies has shown that CRP is associated with plaque formation and rupture; therefore, it has a significant prognostic role for incident and recurrent ACS and other cardiovascular events [49,50,51].

It is also known that patients with depression present altered autonomic tone, which might cause cardiac autonomic dysfunction in depression [52]. Sympathetic overactivity and parasympathetic hypoactivity lead to autonomous dysfunction, causing elevated heart rate and decreased heart rate variability [53,54,55]. While increased heart rate variability (HRV) is met in normal cardiac function, decreased HRV is more frequent in patients with CAD and/or HF [56]. It is noteworthy that HRV is significantly lower in depressed compared with non-depressed patients. Against this background, in a sub-analysis of the ENRICHD study, Carney et al. showed that low HRV mediates the impact of depression on survival after an ACS [57].

Furthermore, the hypothalamic–pituitary–adrenocortical axis might be affected by depression. It is known that depression is associated with higher levels of corticotropin-releasing factor (CRF) in the cerebrospinal fluid and more neurons producing CRF. Considering that CRF increases the levels of corticosteroids into bloodstream, it is reasonably assumed to trigger atherosclerosis, dyslipidemia, hypertension, hyperglycemia, and hypertriglyceridemia [40,58].

Additionally, the role of platelets in ACS and CCS has been well-documented. Hyperactivation of platelets leads to the formation of intracoronary thrombi, which is the most typical mechanism of ACS [59]. Interestingly, major depression has been associated with increased platelet activation [60]. Moreover, Berk and Plein [61] studied the response of intracellular calcium to thrombin stimulation. Intracellular calcium is a second messenger in platelet aggregation and is associated with thrombin simulation. Considering that patients suffering from major depressive disorder have shown heightened sensitivity to thrombin stimulation, platelet intracellular calcium response to thrombin stimulation might mediate in the pathophysiology of depression and CAD [61].

Lately, the role of endothelial dysfunction has gained ground in explaining multiple pathophysiological mechanisms of CVD. Sherwood et al. studied 143 patients with documented CAD, by performing flow-mediated dilation of the brachial artery, a reliable index of endothelial function. Patients with depression had decreased flow-mediated dilation compared with these without, and treatment with antidepressant drugs was related to better flow-mediated dilation [62].

Depression and CAD might share common genetic pathways. While depression seems to be hereditable and individuals with a first-degree relative diagnosed with depression have an about three-fold higher risk of facing depression, several genome-based studies targeting the detection of frequent genetic variants that contribute to the development of depression have failed to identify any consistent genetic variation [25,63]. Depression and CAD might not only have a bidirectional link, but they might also be provoked by the same genetic pathway. More specifically, a total of 192 genes that have been associated with both development of CAD and depression have been identified [64].

Apart from molecular and pathophysiological mechanisms connecting CAD and depression, there are other profound reasons why patients with depression face a higher risk of CAD developing and have poorer prognosis. It is well-established that patients with depression have an unhealthy lifestyle, like tobacco smoking, drug use, lack of exercise, an unhealthy diet, and excessive alcohol drinking; all these parameters are significantly associated with higher risk of acute MI and CAD [65]. Additionally, depressed patients have poor medical compliance, which plays a vital role on the primary and secondary prevention for cardiovascular diseases [66].

Sex-based differences in the interplay between depression and CAD are consistently observed in epidemiological and clinical studies. The reasons explaining this gender discrepancy remain unknown. It might be associated with early life adversities and psychological trauma during childhood, which occurs more frequently in girls than boys and may affect neurohormonal development. Such conditions could prepare the ground for developing CAD later in girls’ and women’s lives [67]. The underlying mechanisms for these disparities extend beyond psychosocial dimensions and are increasingly understood to involve sex-specific neurohormonal and autonomic regulation. Early-life adversities, including childhood trauma and abuse—which occur more frequently in females—have been implicated in long-term dysregulation of the hypothalamic–pituitary–adrenal (HPA) axis. Such dysregulation results in sustained elevations in cortisol and corticotropin-releasing factor (CRF), both of which contribute to systemic inflammation, endothelial dysfunction, and metabolic derangements that promote atherogenesis. Moreover, current evidence supports the role of estrogen in modulating both cardiovascular and neuropsychiatric resilience. Estrogen enhances endothelial function, promotes vasodilation using nitric oxide pathways, and has anti-inflammatory and antioxidative effects. In the central nervous system, estrogen interacts with serotonin and dopamine systems, modulates stress reactivity, and may protect against hippocampal atrophy. The loss or attenuation of these protective effects—due to premature ovarian insufficiency, hormonal contraceptive use, or fluctuating estrogen states—may amplify susceptibility to both depression and CAD [68,69]. Pizzi et al. further demonstrated that young women with MI and comorbid mental stress ischemia exhibited heightened autonomic imbalance, characterized by low heart rate variability and elevated peripheral resistance—findings not observed in similarly aged men. This suggests that the autonomic consequences of psychological stress may be more deleterious in women, particularly in the presence of antecedent trauma [70].

Emerging evidence suggests that microvascular dysfunction may serve as a key pathophysiological link between late-life depression and cardiovascular disease. A systematic review and meta-analysis by van Agtmaal et al. demonstrated that depression in older adults is associated with markers of systemic and cerebral small vessel disease, including impaired endothelial function, reduced cerebral perfusion, and white matter hyperintensities [71]. These findings imply that microvascular injury might not only contribute to the cognitive and affective symptoms of late-life depression, but also present a wider vascular vulnerability that includes the coronary microcirculation. This is particularly relevant given growing recognition of coronary microvascular dysfunction (CMD) as a distinct clinical entity in patients, and especially women, presenting with angina and non-obstructive CAD [72]. Chronic systemic inflammation, endothelial dysfunction, and autonomic imbalance may act as shared contributors to both CMD and cerebrovascular compromise, linking depressive symptoms with ischemia in both organs. In parallel, angina with nonobstructive coronary arteries (ANOCA)—a syndrome increasingly recognized in middle-aged women—is strongly associated with CMD and autonomic imbalance, which are also implicated in the neurobiology of depression. Patients with ANOCA often experience recurrent, unexplained chest pain, despite non-obstructive coronary findings. This diagnostic ambiguity contributes to frustration, stigmatization, and under-treatment, all of which are known drivers of depressive symptomatology. Studies report that individuals with ANOCA experience higher rates of anxiety and depression, and a diminished quality of life compared with those with obstructive CAD [73,74,75]. The overlap of functional ischemia, systemic microvascular pathology, and emotional distress highlights the need for integrated care pathways and suggests that targeting microvascular health—via both cardiovascular and psychiatric strategies—could offer dual benefit in these high-risk populations.

**Table 1:** Overview of pathophysiological mechanisms linking depression and coronary artery disease.

Mechanism	Biological Pathway	Supporting Evidence
Sympathetic hyperactivity	Increased norepinephrine release → elevated heart rate, reduced HRV, arrhythmias.	Elevated urinary catecholamines in depression; low HRV predicts post-MI mortality [6,52,57].
Chronic inflammation	Pro-inflammatory cytokines (IL-6, TNF-α, CRP) → endothelial dysfunction, atherosclerosis.	CRP levels correlate with depression severity and ACS risk [49,50,51].
Platelet hyperactivation	Increased thrombin sensitivity → thrombus formation in coronary arteries.	Depressed patients show increased platelet aggregation [40,60,61].
HPA axis dysregulation	Elevated cortisol → hypertension, dyslipidemia, insulin resistance.	CRF overexpression → atherosclerosis [40,58].
Autonomic dysfunction	Sympathetic overactivity + parasympathetic hypoactivity → cardiac instability.	Low HRV mediates depression’s impact on post-ACS survival [52,57].
Shared genetic pathways	Overlapping genes (e.g., inflammatory, neurohormonal) predispose to both conditions.	192 genes linked to both CAD and depression [64].

## 5. Management

Management of CAD patients with depression needs a holistic approach from different medical specialties. Psychotherapy remains the cornerstone of psychiatric, non-pharmacological treatment, as it helps people suffering from depression to understand the behaviors, feelings, and ideas associated with their negative emotions. CBT remains the main psychotherapy approach, by exploring thought patterns that might be undesirable and self-defeating [11]. Psychotherapy has been shown to be equally effective as pharmaceutical treatments for depression, and even more efficient in some specific patients. However, it remains unknown whether psychotherapy would reduce mortality rates in patients with CAD; some studies supported that psychotherapy has been associated with a 70% reduction while others did not support this [37,76,77]. In the ENRICHD study, CBT failed to show benefit compared to standard medical treatment regarding patients’ prognosis, although it positively affected their psychology [78].

Nowadays, selective serotonin reuptake inhibitors (SSRIs), like paroxetine, escitalopram, fluoxetine, citalopram, fluvoxamine, and sertraline, are considered as the gold- standard treatment for depression. (Table 2) Although they are first- line treatment, they show only modest efficacy, as 80% of improvement is associated with a placebo effect and they are most effective in patients with major depression. They are preferred due to having few or no anticholinergic adverse effects. Their favorable profile is closely associated with their action mechanism; they act by blocking the transporter that returns the serotonin from the synapse into the neuron [11]. Therefore, they are a first line of treatment for CAD patients. Although they are safe, it remains unclear whether they improve cardiac function per se. For example, the SADHART (Sertraline Antidepressant Heart-Attack Randomized Trial) was a randomized, double-blind, placebo-controlled trial, investigating whether sertraline helped in-hospital patients with ACS and depression. Although sertraline improved the symptoms of depression, it failed to significantly reduce major adverse cardiovascular events [79]. Despite having a safer profile than tricyclic antidepressants (TCAs), administration of SSRs does not come without a cost; high doses have been associated with important QTc lengthening and ventricular arrhythmias [80,81]. Additionally, SSRIs have an antiplatelet activity and act synergistically with antiplatelet drugs, as they inhibit the uptake of serotonin by platelets and block their activation; therefore, they increase bleeding risk, especially in patients treated with an antiplatelet agent (aspirin and/or P2Y12 inhibitor) or oral anticoagulation [11,39]. Generally, sertraline should be preferred over other antidepressant drugs in patients with a recent MI, as it is the most studied one. On the other hand, citalopram should be avoided or used with caution in patients at high risk of QTc prolongation or Torsades de Pointes, like those with HF, recent MI, bradycardia, electrolyte imbalance, or congenital long QT syndrome.

Serotonin–norepinephrine reuptake inhibitors (SNRIs), such as duloxetine, venlafaxine, and levomilnacipran, have been introduced to clinical practice. They inhibit re-uptake of both serotonin and norepinephrine from pre-synaptic neurons. Considering that they not only block serotonin, like SSRIs, receptors, and norepinephrine, it is reasonable that they are more effective antidepressants, but with more adverse events. Venlafaxine has been linked with a dose-dependent increase in blood pressure, which should be avoided in patients with CAD, especially those suffering from hypertension, with a three-fold increased risk of attempted or completed suicide [82]. Despite this theoretical increase in BP, a comparative study of venlafaxine and sertraline in patients with CVD did not reveal any difference in adverse events; nevertheless, venlafaxine was associated with lower incidence of heart failure [11,83,84]. Duloxetine and levomilnacipran, other members of the SNRI class, have not been sufficiently studied in this specific population to support reliable conclusions. Their use should be considered cautiously and on a case-by-case basis, with attention to comorbid hypertension and arrhythmic risk.

Tricyclic antidepressants, such as amitriptyline, imipramine, desipramine, nortriptyline, and clomipramine, are the older agents and, to date, they have been used for the treatment of major depression. Their mechanism of action is based on the inhibition of the uptake of presynaptic neurotransmitters (norepinephrine and serotonin) and their increase into the bloodstream. Although they were used extensively between 1950 and 1980 for the treatment of depression and other major psychiatric disorders, their significant side effects decreased their usage in clinical practice [85]. More specifically, they are associated with prolongation of the PR and QTc interval and QRS duration flattening of the T wave, which theoretically could cause ventricular arrhythmias and even sudden cardiac death [80]. Therefore, they should generally be avoided in the majority of patients suffering from CVDs such as CAD, abnormal conduction, and congestive HF. TCAs are especially contraindicated in the treatment of patients with a recent MI. We should highlight that for patients having a major cardiac event (i.e., MI) or who have experienced adverse events from TCAs, a slow tapering before stopping is mandatory, as abrupt withdrawal has been associated with an increased risk of malignant arrhythmias [11,84].

There are several other antidepressants that are not included in the previous categories and their exact mechanisms of action are not clear. Bupropion, a norepinephrine–dopamine reuptake inhibitor, is unique in its utility for both depression and smoking cessation. It is generally well tolerated from a cardiovascular perspective, with data suggesting neutrality regarding QTc prolongation and arrhythmic risk. Its use may be particularly advantageous in CAD patients with comorbid nicotine dependence. However, it should be avoided in individuals with a history of seizures or recent myocardial infarction, where increased sympathetic activation may pose a risk [86,87]. Mirtazapine, an α2-adrenergic antagonist that increases noradrenergic and serotonergic transmission, is often reserved for patients with insomnia or appetite loss. Despite its generally low arrhythmic risk, it has been associated with weight gain and, when combined with clonidine, may precipitate hypertensive crises. Limited data in cardiovascular populations preclude routine use [88]. Trazodone, a serotonin antagonist and reuptake inhibitor, is frequently prescribed off-label for sleep disturbances. While typically well tolerated in low doses, it has been associated with QTc prolongation, particularly in overdose or when co-administered with antiarrhythmic agents such as amiodarone. Caution is warranted in elderly patients or those with baseline conduction abnormalities [89,90,91].

Sodium–glucose co-transporter 2 (SGLT2) inhibitors, such as empagliflozin and dapagliflozin, have demonstrated substantial benefits in heart failure, chronic kidney disease, and atherosclerotic cardiovascular disease, irrespective of diabetic status. Recent experimental studies have also uncovered a potential antidepressant effect of this drug class. A preclinical study showed that SGLT2 inhibition led to elevated brain-derived neurotrophic factor (BDNF) levels in key brain regions associated with mood regulation, including the hippocampus. BDNF plays a central role in neuronal survival, neurogenesis, and synaptic plasticity—mechanisms that are commonly disrupted in major depressive disorders. These findings suggest that SGLT2 inhibitors may have central neurotrophic effects that extend beyond their hemodynamic and metabolic profiles [92]. While clinical data are still limited, ongoing trials are investigating mood-related outcomes in patients treated with SGLT2 inhibitors, especially those with comorbid cardiovascular and psychiatric conditions. If validated in humans, this class may represent a novel therapeutic bridge between cardiology and psychiatry, offering both vascular protection and mood stabilization in high-risk populations.

**Table 2:** Pharmacological treatment options for depression in CAD patients.

Drug Class	Examples	Efficacy in CAD	Cardiac Risks	Guidelines
SSRIs	Sertraline, citalopram	Sertraline: safe post-MI; improves depression (SADHART trial). Citalopram: avoid in QTc prolongation.	QTc prolongation (citalopram), bleeding risk (antiplatelet synergy).	First-line per ESC/AHA; prefer sertraline for safety [79,81].
SNRIs	Venlafaxine	Modest efficacy; may increase BP (dose-dependent).	Hypertension risk; limited evidence for CAD-specific harm [82].	Second-line; monitor BP in hypertensive patients [11,84].
TCAs	Amitriptyline	Avoid in CAD: arrhythmogenic (prolonged QTc), contraindicated post-MI.	High cardiac toxicity; sudden-death risk [80,85].	Not recommended in CAD [11,84].
Atypical Agents	Bupropion	Useful for smoking cessation; neutral cardiac profile.	Minimal cardiac effects; safe in stable CAD [86,87].	Preferred for comorbid CAD and smoking [86,87].

## 6. Future Directions

The bidirectional relationship between CAD and mental illnesses presents a critical avenue for interdisciplinary research, with several unresolved questions and opportunities for innovation and progress. Mechanistic studies should prioritize exploring the functional roles of mutual genetic pathways, particularly the 192 genes existing in both conditions, to identify novel therapeutic targets. Epigenetic investigations, such as DNA methylation patterns linked to chronic stress or inflammation, could also unravel how environmental factors modify this comorbidity [93]. Moreover, the gut–brain–heart axis remains under investigation; understanding how microbiota dysbiosis in depression influences systemic inflammation and endothelial dysfunction might help us develop prebiotic or probiotic adjunct therapies [94]. Mitochondrial dysfunction, which is a potential bridge between depressive pathophysiology and ischemic injury, should be also explored further, particularly regarding oxidative stress and bioenergetic failures. Picard et al. demonstrated that psychological stress induces mitochondrial fragmentation and altered respiratory chain activity in both neurons and cardiomyocytes. These findings support ongoing research into mitochondrial-targeted therapies—such as Coenzyme Q10, nicotinamide riboside, and mitophagy enhancers—as potential adjuncts in psychocardiology [95].

Clinically, personalized medicine approaches must utilize biomarker-driven strategies—such as inflammatory markers, platelet activity, and genetic risk scores—to identify high-risk populations and develop relevant interventions. Pharmacogenomic studies are required to optimize antidepressant drug selection in CAD patients, balancing efficacy (e.g., SSRIs like sertraline) with cardiac safety, especially in patients with high arrhythmic risk. Emerging pharmacogenomic data may inform antidepressant selection in patients with heightened arrhythmic risk. CYP2C19 and CYP2D6 polymorphisms, for instance, influence the metabolism of SSRIs, and their genotyping could guide safer, more effective prescribing.

Additionally, sex-specific research is critical in addressing the gender disparity; could hormonal factors, like estrogen’s role in neuroprotection and vasodilation and psychosocial stressors unique to women affect the developing therapies?

Furthermore, technological innovations, like AI-driven predictive models using EHR data, could enhance early detection of depression in CAD cohorts or predict cardiovascular events in populations with depressive disorder [96]. Digital therapeutics, including app-based CBT and wearable devices monitoring HRV, may improve adherence and real-time management [97]. Socioeconomic integration demands scalable solutions, such as telemedicine platforms and collaborative care networks, to overcome accessibility barriers in low-resource settings. Policy reforms should advocate for universal depression screening in cardiac rehabilitation programs and insurance coverage for integrated care models. Furthermore, collaborative care models, which embed psychiatric evaluation and treatment within cardiovascular clinics, have demonstrated reductions in mortality and rehospitalization. A meta-analysis of such models showed a significant reduction in all-cause mortality among CAD patients with comorbid depression, compared to usual care [98]. Broader implementation of these models—especially in resource-limited settings—requires policy support, scalable workflows, and interdisciplinary training.

Finally, global health initiatives must prioritize regions where urbanization and lifestyle shifts exacerbate CAD–mental health burdens, yet remain understudied [99]. Last but not least, the use of anti-inflammatory agents and exploring the application of neuromodulation techniques might be effective in patients with refractory symptoms.

## 7. Conclusions

The interplay between CAD and depression presents a complex, bidirectional link, with each condition increasing the risk and progression of the other. Depression significantly impacts adherence to pharmaceutical treatment, lifestyle modifications, and overall prognosis in patients suffering from CAD; on the other hand, CAD might provoke depressive symptoms through mutual biological pathways, including inflammation and neurohormonal dysregulation. Recognizing and managing depression in CAD patients is vital for improving clinical outcomes. Multidisciplinary approaches incorporating mental health assessment and pharmaceutical and non-pharmaceutical interventions alongside standard cardiovascular care are critical. Future research should focus on optimizing integrated care models and elucidating the mechanisms underlying this interconnected relationship.

## Data Availability

Available upon request from corresponding author.
